# Editorial: Soil microbes in polar region: Response, adaptation and mitigation of climate change

**DOI:** 10.3389/fmicb.2022.1086822

**Published:** 2022-12-06

**Authors:** Gang Yang, Jianqing Tian, Ji Chen

**Affiliations:** ^1^School of Life Science and Engineering, Southwest University of Science and Technology, Mianyang, China; ^2^Institute of Botany, Chinese Academy of Sciences, Beijing, China; ^3^Department of Agroecology, Aarhus University, Tjele, Denmark; ^4^Aarhus University Centre for Circular Bioeconomy, Aarhus University, Tjele, Denmark; ^5^iCLIMATE Interdisciplinary Centre for Climate Change, Aarhus University, Roskilde, Denmark

**Keywords:** global warming, microbial community, terrestrial ecosystem, polar region, soil microbial

Soil microbes govern the biogeochemical cycling of elements. Understanding and predicting the impacts of climate warming on soil microbes present a great challenge (Jansson and Hofmockel, 2020). Increasingly, studies in microbiology are investigating the responses and feedback of soil microbes to global warming and environmental changes (Boetius, 2019). Regions that are particularly sensitive to climate change, for example, Antarctica, the Arctic, the Qinghai-Tibet Plateau, and high alpine regions generally, are the best places to study the response of microbial communities to climate warming (Chen et al., 2022). For example, by synthesizing 110 observations from 64 experimental warming studies, Chen et al. (2016a) showed that warming generally had stronger effects on soil microorganisms in colder regions. Apart from climate warming, the polar regions are also experiencing unprecedented intensifying nitrogen deposition and water regime changes (Jansson and Hofmockel, 2020; Luo et al., 2020). In recent years, numerous studies carried out in the polar regions have demonstrated that soil microbial diversity, community structure, and abundance were significantly correlated with environmental biotic and abiotic parameters, including water regime, soil temperature, and nitrogen deposition (Chen et al., 2016b). With an improved understanding of the importance of microorganisms in the polar regions, we can uncover how microorganisms respond to climate and environmental change, and mitigate these factors. This Research Topic, entitled “Soil microbes in the polar regions: Response, adaptation, and mitigation of climate change” comprises five original articles on soil microbes in Polar regions, which were contributed by 34 authors.

As climate change factors, nitrogen (N) and phosphorus (P) deposition have substantially increased over the past century, and have profoundly impacted various ecosystems. Cao et al. study the microbial response to short- (2 years) and long-term (10 years) nitrogen and phosphorus additions in peatland ecosystems, characterized by low nitrogen and phosphorus availabilities. The study finds that short- and long-term fertilization changed the abundance of plant functional types, but there were no cascading effects on fungal community structure. On the other hand, strongly contrasting effects on fungal community composition and diversity were observed after short- and long-term nutrient additions. Long-term nutrient addition reduced *Sphagnum* coverage and relative abundances of mycorrhizal fungi but increased the relative abundance of lignocellulose degrading fungi. This study demonstrates that the cascading effects of altered plant functional types on soil microorganisms in peatlands probably take a longer time to show up.

The effects of climate and land use changes on soil aggregate-associated carbon through regulating Arbuscular mycorrhizal fungi (AMF) are complex processes and poorly investigated. Yang et al. conduct an experiment to simulate climate and land use changes from cropland to fallow land on soil AMF and aggregate-associated carbon. The results show that the diversity and the network complexity of the AMF community in fallow land were higher than in cropland. AMF communities have a positive relation with mean annual temperature and precipitation. The amounts of soil aggregate-associated organic carbon were significantly higher in long-term fallow land than that in cropland, due to the higher hyphal length density, and increased glomalin-related soil proteins, mean weight diameter, and geometric mean diameter of AMF in fallow land. This study elucidates the significance of AMF as a carbon sink for climate warming mitigation.

A plant's rhizosphere forms a reservoir for soil microbes, and its structure and diversity can reflect ecosystem function. Fu et al. use high-throughput sequencing to study microbial communities in rhizosphere soil and their response to different grassland management approaches (mowing, grazing, and enclosing) in northern Tibet. Rhizosphere soil microorganisms showed no significant differences in relative abundance at phylum and genus levels. *Proteobacteria* and *Actinobacteria* were the dominant bacteria in rhizosphere soil and represented the core species of microbial networks in the alpine grassland of northern Tibet that stabilized the microbial communities. These valuable findings will benefit the restoration of degraded alpine grasslands.

Warming and water scarcity as the two main factors of climate change that significantly affect soil microbial community and structure (Song et al., 2021; Xue et al., 2021). Two studies by Li W. et al. and Li M. et al. focus on microbial effects of soil water content and soil warming in the degrading peatlands and wetlands of the Qinghai-Tibetan Plateau. Li W. et al. investigate the response of methanogens to 1-year short-term warming, drought (20%), and their combined effects in the degraded peatlands of the Zoige Plateau in China. The results indicate that drought significantly decreased the copy number of methanogens, whereas the 1-year short-term warming had no effect. Li M. et al. study microbial community changes along degradation gradients in the same regions of the Tibetan Plateau and find that wetland degradation from wetland soil to grassland soil did not affect microbial community richness and diversity. However, wetland degradation strongly affected microbial community structure. Soil water content was the key factor that influenced microbial community composition and microbial networks of *Actinobacteriota, Acidobacteriota, Cholorflexi*, and *Proteovacteria*. This study explores how microbial community composition responded to changing soil properties and environmental changes during alpine wetlands degradations.

In summary, the articles included in this Research Topic demonstrate the effects of climate change factors, including nitrogen and phosphorus enrichment, climate warming, water gradients, and land use changes on soil microbial community structures in high-latitude regions and the Qinghai-Tibetan Plateau of China. The results reveal how soil microorganisms respond to aspects of climate change including changing vegetation, aggregates, temperature, and moisture, and thus enrich our knowledge of soil microorganisms in the polar regions.

## Author contributions

All authors listed have made a substantial, direct, and intellectual contribution to the work and approved it for publication.

## Funding

This Research Topic was supported by the National Natural Science Foundation of China (42077038) and the Sichuan Science and Technology Program (2020YFS0020).

## Conflict of interest

The authors declare that the research was conducted in the absence of any commercial or financial relationships that could be construed as a potential conflict of interest.

## Publisher's note

All claims expressed in this article are solely those of the authors and do not necessarily represent those of their affiliated organizations, or those of the publisher, the editors and the reviewers. Any product that may be evaluated in this article, or claim that may be made by its manufacturer, is not guaranteed or endorsed by the publisher.
